# Valedictory Address to the Graduating Class in Chicago Medical College, Medical Department Lind University, at the Fifth Annual Commencement, March 3, 1864

**Published:** 1864-04

**Authors:** Henry Wing

**Affiliations:** Prof. of General Pathology and Public Hygiene


### CHICAGO MEDICAL EXAMINER.

N. S. DAVIS, M.D., Editor.

VOL. V. APRIL, 1864. NO. 4.

### Original Contributions.

ARTICLE XIII.

### VALEDICTORY ADDRESS TO THE GRADUATING CLASS IN CHICAGO MEDICAL COLLEGE, MEDICAL DEPARTMENT LIND UNIVERSITY,

*At the Fifth Annual Commencement, March* 3, 1864.

By HENRY WING, M.D., Prof. of General Pathology and Public Hygiene.

Gentlemen of the Graduating Class:—

It has been truly said, that there is but one journey through life, and no going back to correct mistakes.

One of the most impressive passages in all profane literature is repeated day by day in our schools, and no one thinks of its meaning. It is in these words: "I might have been, thou mightst have been, he might have been."

In the lips of those who utter the words there, they have no meaning, because all their possibilities are in the future. But the words in their significant use imply a possibility or a power which once existed and now exists no more—capacities, that for those who speak, will, like the raven in the immortal poem, live only in the undying echoes of "nevermore."

Such is the nature of life. Each opportunity comes but once. To the young all paths are open. To the aged the way is fenced up and hedged in, by habit, by talents developed, and talents wanting, and to a certain extent, man has become the prisoner of his own faculties, so that new ways, and new thoughts, and new activities are difficult, and even impossible to him.

And, as it is true that as a man thinketh so is he, so as a man *does*, so will he think; that is, habitual occupation molds the character.

In life these influences are gradual and stealthy. So it is that by degrees a profession wears into a man, and what once would have seemed to him a quaint, odd picture, is his own likeness. If the picture were presented to him before he had grown into it, perhaps he would regard it with great repugnance, and he would say with BENEDICT in the play:—

"Can *I* be so converted, and see with these eyes?"

Such being the influence of life, it is well that in the course of it there are, as it were, landing-places to the stairs, where we are invited to pause and look around—to digest what has gone before, and survey the different prospects which different courses offer in the future.

Such a point, gentlemen, is this in your history. After long and laborious study, you have received the approbation of those who have been authorized by the high authority of your State, to give it, to your entering an honorable profession, which it is presumed you will pursue for life.

But as yet you have not entered upon your professional course. Life is all before you, and you are free. Notwithstanding the time you have spent in the preparation, you are under no compulsion to go forward. All that time has been employed in acquiring a knowledge of man and of his relations to things around him.

Multitudes of thinkers have approved the declaration that

"The proper study of mankind is man."

Whether we accept the statement or not, there can be no harm in spending a portion of our early years in that study, whatever may be our future employment.

Let us, then, in a spirit of candor, examine the Profession of Medicine, remembering that, if we make a mistake, there is no going back, after we arrive at the end, to correct it.

First, we look at it as a means of Intellectual Culture.

Different studies have their advocates as a means of cultivating the powers of the mind. And we know that some studies tend to develop the mind unequally, if too exclusively pursued. A man may study mathematics exclusively until his mind runs by strong habit, in the channel of numbers and quantity, and his apprehension of other subjects is enfeebled.

Again, some studies, exclusively pursued, diminish one's *relish* for other subjects. Some men become so devoted to collecting specimens of some single department of natural history, that they take little interest in the great living and moving world. Some such would rather see a specimen of a new beetle, than Lake George or Mount Washington. To them, the question of rank betweea a lobster and a crab is a matter of very grave importance, while they do not care whether HALLECK outranks GRANT, or GRANT HALLECK.

Such men have their uses, of course. We may well thank them for their labors, but we can hardly regard them as symmetrical characters, or their experience as one to be chosen as the most desirable.

I need not tell you that the pursuit of Medicine is no such exclusive subject. There was a time, perhaps, when you shared the impression of many others, that it was peculiar and limited, that a Doctor's brain was something like an old garret, stored with things out of the way of common affairs. That he mused upon pains and aches, pills and powders, and disgusting draughts.

But your first introduction to the subject showed you that it was to be approached through sciences commonly regarded as altogether foreign to man.

You have seen how the elements of man's nature and the laws of their action, must be sought for outside of his body, in other objects and structures. To be understood they must be taken in their simplest forms. And the study of those simple forms leads you to the study of all inorganic substances and forces, and of all vegetable and animal life. The water of the ocean, the oxygen which forms so large a part of the mountain, must have no law of action which is unknown to you. In the whole range of vegetable and animal life, there must be no organization—no function, the law of which you do not understand, in at least some of its uses. In the simplest organizations you learn the alphabet of the science of man, and step by step advance to the higher, until you reach the most complex and mysterious organization, which, in some degree, represents all the rest.

If not directly a student of all the physical and natural sciences, you are at least made acquainted with their results; and habit and association invite you to the investigation of that "high history" of man which involves the geological development of his place of abode, and all the elements and forms which coëxist with him. The study of disease is but a more extended and critical study of the conditions of life.

Neither is this quite all. You are concerned with man's social relations, employments, and pleasures, and faith for all these bear largely upon that health which is your especial concern. To some degree, then, you are students of institutions, of customs, and of the passions of men.

We have, then, a study of the most liberal character—wide as the world itself—deep as human life—related to, if it does not include, all the creations of God.

If you pursue this great subject in the right spirit, you will tread reverently along the path where the Creator has gone in His work, leaving behind him the record of His thought, and your whole professional study will be a walk in His temple.

There is, however, some apprehension growing out of the very breadth of the subject. There are some men who despair of keeping up with the profession, amid the multiplied cares of life, and conclude that as they have done well they can continue to do so on what knowledge they have. I warn you that if you ever indulge such a thought, it will be fatal to your intellectual culture; and it would be far better to be a shoemaker, devoting your sedentary hours to reflection, than practicing an art which you are conscious you do not fully understand; for all who do not go forward must go backward.

But the Practice of Medicine (somewhat like Morals) is different from the study. The influence upon character comes quite as much, perhaps, from that source.

It is at once apparent that the life of the Physician is not altogether natural; that is, a good part of it is among scenes not altogether like life in general. He is so much amidst pain, and suffering, and bereavement, that it might be supposed that he would ultimately lose some of the finer sensibilities of his nature.

When a great epidemic forces men to live in the atmosphere of suffering and sorrow, multitudes shield themselves from the unwelcome experience, by refusing to feel anything of what they see. And the capacity of indifference is cultivated so rapidly that it startles us in the contemplation, and almost makes us doubt whether man be not really something worse than a beast.

There are some, perhaps, upon whom the experience of the profession has a similar influence. Men who began their professional life with no proper conception of its obligations, and adopting the sentiment,

"The world is mine oyster."

Such a man bends over a tumor with a keen, practiced eye, and hard, coppery face, to see how much money there is in it. Or how much renown, to yield money at last, might be made by excising it, and publishing an account of the successful operation, omitting to state that the patient died of the effects of the operation.

Such a man, if he cultivates science as he generally does, pursues it for ulterior ends, and without pleasure in the subject itself. In the fields he is no guest of Nature, but a

——"Fingering slave,
One who would peep and botanize
Upon his mother's grave."

Surgeons are more exposed to become indifferent to the feelings of their patients, because they have but little acquaintance with them, and if they have a large practice, they are almost compelled to look upon them as subjects for operations merely.

The cause most likely to lead the physician in that direction is neglect of study. The physician who neglects study, necessarily becomes empirical and dogmatical. He does not preserve clear ideas of the causes of the phenomena with which he deals. Thus he comes to look upon pain and suffering as a blind fatality, and shuts his ears to its cry with a half recognized but smothered consciousness of deficiency, which is not far removed from guilt. But the physician who keeps pace with the progress of his profession, knows that he labors daily to understand more fully the laws of that suffering which he is unable to relieve. And in the study even of cancer, and those diseases which are the most fearful to contemplate, our labor is rewarded by the acquisition of increased ability to palliate what we cannot cure. Moreover, even in such diseases, we find some play of beneficent laws, a seeming effort of nature to do that which is best, and we feel that even there, there "is a spirit of good in things evil," which exhorts to kind feeling and beneficent action.

But there is a counterpart even to this view—a bright side to the dark picture. It may fairly be assumed that some, at least, of our cases, would not be fatal. If a physician is truly skilful, it is his constant privilege to give relief to suffering, and not infrequently to prolong lives of inestimable value. We do not desire here to magnify our powers. We owe it to you on this occasion to be candid. We know that there are manifold difficulties, many uncertainties, and painful limitations to our knowledge. And yet it is true that the power which the skilful physician holds in his hand is, in many cases, very great indeed. Well may he rejoice in it as more wonderful and more to be coveted than the fabled lamp of Aladdin. The physician who does not feel a high enthusiasm over the power of his art, and feel profoundly grateful for it, does not know its resources, and is unworthy to pursue it.*

If, then, the Physician is true to his calling, he not only sees more suffering than other men, but he does more than any other

*[The profession has been depreciated because of the diversity of counsel given to THEODORE PARKER for an incurable malady. Farmers differ as much as to what grain to sow, what manure to use, and how to cultivate, but the grain houses of Chicago testify that they do raise corn with some success.]

to relieve it, and his sympathies grow in the exercise, and in proportion as they grow, yield the more pleasure in their gratification.

But if the associations of the physician are peculiar in the respect that we have just considered, in others, they are the most catholic of all vocations. No rank, no sect, no party, is excluded from his acquaintance. There can scarcely be anything in life, or in human experience, that he does not know. His knowledge and culture make him the companion of the best and wisest men, and his duties make him acquainted with the worst and lowest. Characters that other people only read about are his familiars. Mrs. PARTINGTON is his intimate acquaintance, and he knows that the old lady has many excellent qualities besides her amusing speeches, which are a cure for the "perplexy." He knows CÆSAR in the Senate Chamber, as other men do, and he sees him in the fever when he cries for drink "like a sick girl." As much as possible, he sees the inner life of men when pain has torn off all disguise. Crime that hides itself from society, is open to him; and sorrow that is not spoken to the husband or wife, asks his counsel. He enters the house, not as a heartless critic, or vapid gossip, but as something more than a friend—almost as one of the household.

Woe to the man who abuses such opportunities, and judges severely even in his own mind, though he never speaks it, the life that is thus exhibited to him.

The discipline of the Profession includes some trials in regard to money. It is possible that some other men know something of this subject also, but probably the experience of the physician has its peculiar features. The first trial is before he has made a reputation, and when he has, perhaps, difficulty in sustaining himself while he waits for the slow growth of confidence. He is tempted then to resort to some quackish means of attracting public attention, or to make unwarrantable pretences and promises to his patients. If he yields a little, he receives a stain upon his integrity, and it will require years of struggle to restore his balance of character, and secure his own self-respect. If he goes farther, and becomes a pretender, we will not stop to discuss what becomes of a lost man.

The second trial on the subject of money is perhaps in middle life, when the physician finds that other men, with far less labor and care, and not half the skill, grow rich, while he has only a respectable competency. I give you fair warning that in the practice of medicine, though you have a right to expect to have all that is necessary for the best culture, you probably will never grow rich enough to be shut out, for that reason, from the kingdom of heaven. You may have a neighbor, perhaps, with ten times your income, and made by far less labor. His house will be high and many-chambered, while yours is only neat and comfortable; large mirrors reduplicate the splendors of his parlors—his walls are hung with pictures which he estimates by the thousands which they cost, while yours are sparsely ornamented with a few gems of art. His face is plump with the juices of all good living, while yours is gently furrowed with care.

Do you sigh for JENKINS' income, his fine house, his pictures, his carriage? If so, the same door is open to you. Make money your study, and you can get it. Perhaps you may secure enough to absorb all your attention; and your children will probably think that fine clothes are more than character, gilded frames more than beauty, and houses more than life.

I know that there are some who think that the profession can be made the means of procuring riches, and some of you have heard the advice from high professional authority, that medical services should be rendered solely for the sake of money in every case, and always with the expectation of accumulating a fortune—that beginners in the profession should claim a recognition of their value to society by refusing to exercise their vocation in any case without pay, and regard large fees as the measure of value of their services. We cannot so advise you; nor can we recognize such a standard for our profession. The pretender, the man who has chosen a short road to an unmerited position among men of science, who devotes but little thought to his profession, depreciates his instructors if he discovers the estimate which he himself entertains of his labors; but as such men are really unworthy to be employed at all, their charges are all unjust, and they not unfrequently are the most exorbitant. The services of the true physician, on the other hand, are, often times, above all price. He is not merely the hired servant of his employer. He is his friend in the best sense of the word.

By many, such a view is regarded as fanciful altogether.

The subject is sufficiently important to justify us in examining it.

We do not propose that a doctor shall dine upon gratitude instead of sirloin, nor drink compliments for tea. He should be supported liberally by those who look to him for counsel in matters of health and life; but to place a pecuniary estimate upon *every* professional service is impracticable in itself, and the young man who takes such a rule for his conduct, insisting upon it in every case, will be slow to acquire the skill of an experienced physician; he will not secure the coveted wealth unless he gets it outside of the profession, and he will live in a state of chronic dissatisfaction with the public and his work.

We put it to an objector: Suppose that sickness of an alarming character invades your family circle. Some one that is near to you is in danger, and the shadow of death is in the house. The Physician comes. He studies the intricate problem of the disorder. A very little thing will turn the scale one way or the other. An omission to note the most minute feature of the case will cause a failure in the treatment. Is it enough that the physician looks at the case with a speculating eye? Would you not prefer, by far more than words can measure, that he should feel something of your solicitude to prompt him to the most careful scrutiny and consideration?

Again. Suppose that he does make the case in some degree his own. He studies the case with that deep insight which solicitude only can give—he forgets himself—forgets his sleep—he sees each new danger before it falls, and wards it off, until at last the dreaded blow which threatened to fall upon the house is averted, and health returns again. Can you write down in your ledger just how much money such a service is worth?

If you say that a proper gratitude would in such a case seek some material expression, more than a customary fee, it is true, but such an acknowledgment is an expression of gratitude and not of debt: it is measured by the means of the giver, and not by the price of the service.

Do we in this propose a rule of conduct that is new and unrecognized by other classes of men?

How is it with the citizen who serves his country in the temporary character of a soldier? When the walled fortress frowns with cannon, and bristles with bayonets, and glares with flashes of destruction, what money would tempt men to cross the treacherous ditch, scale the high wall, breast the hard point of the bayonet, and fall upon the determined foe? And yet, thanks to our noble citizenship, when our country asks for such service, men enough for the work always come forward.

Shall we be behind all others in beneficence to society? Shall we alone

> ——————"Coin the heart,
> And drop the blood for drachms?"

Such has not been the practice. But it has often been claimed that physicians, as a class, are behind no other in deeds of magnanimity and beneficence to others.

When any new and strange disease is approaching from abroad, spreading dismay and death wherever it goes, medical men go forward to meet it, and while breathing the atmosphere of death, study the nature of the disorder, and strive to gain a mastery over it.

If we voluntarily abandon that ground for another, in order to increase our gains and "respectability," what shall we say is the pecuniary value of that man's life who, for money, encounters every pestilence that comes? Or shall we take the other alternative, and rejecting the example of others who have gone forward to meet cholera and other terrible destroyers, join the pale-faced crowd that, as the epidemic comes near, betake themselves to flight?

It is better to have a just estimate of the requirements of your position, and expect to meet them cheerfully, or decline the vocation.

Thus we have presented some of the influences which tend to mold the character of the physician, and in regard to which it behooves you to be on your guard. It is a more trying experience than many others, for the moral character, because it makes large demands for magnanimity, which can hardly be denied with indifference merely, for who refuses to respond to them may fall,

> ——"Like Lucifer,
> Never to rise again."

If you are prepared to meet all these trials, and convert them to good, and if such an employment suits your desires, take it with a just estimate of its defects as well as its excellencies.

The field of labor is one which, as nearly as it is possible, engages the faculties in such a way as to give satisfaction to the mind. It always maintains a harmonious relation between the intellect and the emotional character. Satisfactory employment, in which the whole man grows, is in itself a perennial fountain of good. The webbed foot of the bird must press the water, the wing of the eagle must cleave the air, and man is blest when his faculties are well employed. Many occupations exercise the faculties so unequally as to give a sense of weariness, as a fixed posture does to the body; and hence perhaps the majority of men pursue employments which they long to lay aside, in some happier day to come. We cannot say that the practice of medicine is an absolute guaranty against discontent, but it does furnish employment of the highest value, and to a benevolent mind of the most gratifying nature.

The knowledge of the profession is broad and liberal in the highest degree. It ought to render the mind free from those prejudices and misjudgments of men and things which so often disfigure and unbalance the character. No mode of life leaves the mind open to more of the truest pleasures that life can give. No man better than the physician enjoys the sympathy of warm and devoted friends. Few, if any, enjoy better opportunities for exerting a good influence upon others—to strengthen virtue in the hour of its weakness and trial—to brighten faith when darkened, and comfort those who suffer, it may be, without hope.

No life can give, at the close, when nothing remains but its recollections, more memories which are contemplated with satisfaction, and do *not* give a more than dramatic interest to those words, "I might have been."

Custom assigns us the duty to offer you some words of counsel as we relinquish the position of your instructors, and we avail ourselves of the privilege with pleasure, because in our past relation the zeal with which you have sought for knowledge and loved the truth has bound us together.

Take, then, our parting words as the promptings of an interest in your welfare, and a desire to smooth, if possible, your way in life.

1. Having once successfully entered upon professional life, and ascertained your fitness for it, never turn aside from it for any attraction whatever. Do not be tempted by pecuniary speculation. Neither think to divide your allegiance between your profession and some other employment.

If you divide your attention you will divide your strength, and lower your real, if not your reputed standing. In the practice of the profession, if rightly pursued, there is a constant accumulation of experience, knowledge, and judgment, which are elements of power, and render your services of more value in that department of labor than in any other.

And yet, in a country where the people are the guardians of the public welfare, the duties of the citizen are not to be neglected.

The earliest pages of our history are brightened by the example of members of our profession who have abandoned it temporarily for the service of the country. The name of RUSH is alike honored in the annals of medicine, and in the councils of the nation, at a time when the greatest names of a generation were there clustered together. First, too, among the voluntary martyrs to our national liberty, stands the name of another member of our profession, who left its duties first for those of the public councils, and then for the post of a patriot soldier. It is not our province to-night to speak the praise of JOSEPH WARREN. Neither is it necessary to cite such names, to vindicate those of our profession who, in the first hour of our present national peril, grasped the sword and hastened to the tented field. These examples are mentioned merely to qualify the rule proposed, and show that when the public welfare demands, every citizen is called upon to contribute his services to his country in whatever field they may be most useful. But the physician should never seek public life for the sake of the honors connected with it, and never descend to be a mendicant for office.

2. *Secure and cherish a high reputation.* The means which will build up a sound and growing reputation are precisely those which would be dictated by a desire to honor your profession. As another motive for the same conduct, then, I offer you the additional exhortation,

3. *Love your Profession*, be jealous of its honor, and strive to increase its usefulness and influence.

For both of these objects, first of all have self-respect. Character is the power with which a man works among men to do good. The basis of character is self-respect. And the basis of professional character is a consciousness of thorough qualification. To have this, you must always be a student. Not a reader of many books. But you must carefully observe what comes under your notice, know what others think in regard to it, and have an opinion of your own. To be a student of medicine, you must also keep up a communication with the great body of the profession; attend, as far as possible, professional meetings; and strive to contribute something to the common stock of knowledge through professional publications. Every locality will furnish some facts, at least, which, if carefully noted and faithfully reported, will be valuable, and be some return for the great stock of knowledge which the profession has accumulated by ages of observation and study, and which has been free to you, as it is to all.

With the same regard to your own standing, and the honor of your profession:—

4. Avoid as far as possible all controversy with competitors. Never speak of quacks. Treat every true member of the profession as a brother.

*Endeavor to make your talents as useful as possible to mankind.*

Instead of resolving never to work without pay, resolve to work any how, wherever you can do good.

This is the best policy, even from the lowest stand-point. Practice gives experience, judgment, increased knowledge, and it digests what you already know, as you who have been in daily attendance at the clinic know full well. Do not consider the poor, to whom you render gratuitous services, as unworthy of consideration. Charity practice should be a school of manners as well as medicine. If you allow yourself among one class of patients to neglect any of the nicer proprieties of life, it will not fail to react upon your own character, and either detract something from the polish of a true gentleman, which is a necessary part of the qualifications of a true physician, or render the exercise of such demeanor irksome, and a matter of constant care. The best course in this matter is also the best policy. It is to have a nice regard for the feelings of every patient, and cultivate, in every professional relation, sincere kindness, geniality, and urbanity.

It only remains to say the last word, which friends are loth to speak.

We part with you, gentlemen, with more of hope than regret. Your course thus far, in regard to your profession, excites confident expectations of a noble future. While other institutions have tendered you the privileges of the profession on a briefer course of study, and without practical acquaintance with your duties at the bedside, you have magnanimously chosen to devote your time and labor to an extended and thorough course of study, combined with daily clinical experience. By this course you have shown that you preferred to be well qualified for your duties, rather than be in haste to reap their fruits. You have thus contributed in no small degree to establish a high standard for our profession. Your example will contribute to urge, and ultimately to compel, other institutions to provide equal facilities for more extended study, and to add also clinical instruction. These benefits will be extended to numberless others hereafter. To you, then, belongs the honor to-night of having, so far as you have gone, contributed to honor your profession, as well as be honored by it. You will not fail hereafter to continue in the course you have so well begun. Your labors will be felt in the advancement of our science and the elevation of the practice of our art, by the constant infusion into it of beneficent aims. It is, then, with something of just pride, with much of the pleasure of welcome, that I say to you, in the name of my colleagues, your late teachers, Farewell.

